# Prenatal Treatment Prevents Learning Deficit in Down Syndrome Model

**DOI:** 10.1371/journal.pone.0050724

**Published:** 2012-11-29

**Authors:** Maddalena Incerti, Kari Horowitz, Robin Roberson, Daniel Abebe, Laura Toso, Madeline Caballero, Catherine Y. Spong

**Affiliations:** 1 Unit on Perinatal and Developmental Neurobiology, National Institute of Child and Human Development, National Institute of Health, Bethesda, Maryland, United States of America; 2 Department of Obstetrics and Gynaecology, University of Milano-Bicocca, Ospedale S. Gerardo, Fondazione MBBM, Monza, Italy; 3 Department of Obstetrics and Gynecology, University of Connecticut, Groton, Connecticut, United States of America; Radboud University, The Netherlands

## Abstract

Down syndrome is the most common genetic cause of mental retardation. Active fragments of neurotrophic factors release by astrocyte under the stimulation of vasoactive intestinal peptide, NAPVSIPQ (NAP) and SALLRSIPA (SAL) respectively, have shown therapeutic potential for developmental delay and learning deficits. Previous work demonstrated that NAP+SAL prevent developmental delay and glial deficit in Ts65Dn that is a well-characterized mouse model for Down syndrome. The objective of this study is to evaluate if prenatal treatment with these peptides prevents the learning deficit in the Ts65Dn mice. Pregnant Ts65Dn female and control pregnant females were randomly treated (intraperitoneal injection) on pregnancy days 8 through 12 with saline (placebo) or peptides (NAP 20 µg +SAL 20 µg) daily. Learning was assessed in the offspring (8–10 months) using the Morris Watermaze, which measures the latency to find the hidden platform (decrease in latency denotes learning). The investigators were blinded to the prenatal treatment and genotype. Pups were genotyped as trisomic (Down syndrome) or euploid (control) after completion of all tests. Statistical analysis: two-way ANOVA followed by Neuman-Keuls test for multiple comparisons, P<0.05 was used to denote statistical significance. Trisomic mice who prenatally received placebo (Down syndrome -placebo; n = 11) did not demonstrate learning over the five day period. DS mice that were prenatally exposed to peptides (Down syndrome-peptides; n = 10) learned significantly better than Down syndrome -placebo (p<0.01), and similar to control-placebo (n = 33) and control-peptide (n = 30). In conclusion prenatal treatment with the neuroprotective peptides (NAP+SAL) prevented learning deficits in a Down syndrome model. These findings highlight a possibility for the prevention of sequelae in Down syndrome and suggest a potential pregnancy intervention that may improve outcome.

## Introduction

Down Syndrome (DS) is the most common genetic cause of mental retardation due to triplication of all or part of chromosome 21 [1] and occurs in 1 in 800 live births. In the newborn period, infants with DS have hypotonia and delay in achievement of developmental motor [2] and sensory milestones [3], [4]. At the neuropathological level, neonates with DS have a smaller brain, delayed myelination of neurons and glial alterations [5]. These early developmental anomalies may predispose to abnormalities in adulthood including mental retardation and early onset of Alzheimer disease.

The Ts65Dn mouse is a well-characterized model for DS [6], with triplication of a segment of chromosome 16 which includes over 55% of the genes present on human chromosome 21.

In the newborn period, Ts65Dn mice mimic the human condition, including developmental delay [7]. Microscopically, Ts65Dn neonates have fewer granule cells in the hippocampus, reduced Long Term Potentiation (LTP) and abnormal synaptic plasticity [8]–[10]. In adulthood, Ts65Dn mice have a deficit in short and long term memory, deficits in learning, and early onset of the neuropathology of Alzheimer disease [7], [11]–[14]. The mechanisms underlying the neuropathology of DS are not well understood.

Vasoactive Intestinal Peptide (VIP) is a neuropeptide crucial for the development of the brain [15], [16]. In human studies the level of VIP is increased in neonatal blood of children with DS [17], [18]; Ts65Dn mice have elevated brain VIP mRNA [19], and cortical astrocytes from postnatal day 8 brains show a defect in the signal transduction mechanism of the VIP receptor VPAC-1 with astrocyte dysfunction [20]. Furthermore, blockade of VIP during embryogenesis is followed by postnatal hypotonia, growth restriction and developmental delay [21] in both human DS and Ts65Dn mice. It is hypothesized that the up-regulation of VIP in DS may be an attempt to compensate for the loss of neuronal function [19], which may explain the high levels of VIP [17], [18].

VIP stimulation of astrocytes results in the release of neurotrophic factors, including Activity Dependent Neuroprotective Protein (ADNP) and Activity Dependent Neurotrophic Factor (ADNF), which have been demonstrated to be neuroprotective [22]. Active fragments of ADNP and ADNF, NAPVSIPQ (NAP) and SALLRSIPA (SAL) respectively, have shown therapeutic potential for developmental delay [23], [24] and learning deficits [25], [26]. Addition of SAL or NAP to DS cortical neurons resulted in a 2-fold increase in neuronal survival as well as a reduction of degenerative morphological changes [27].

To date, there is no therapy for the prevention of developmental delays in DS (MEDLINE from 1963, keywords: Down syndrome, treatment, development, fetus; all languages). Our hypothesis was that prenatal treatment with NAP+SAL may prevent the learning deficit in the Ts65Dn mouse model for Down syndrome.

## Materials and Methods

Pregnant Ts65Dn females were randomly assigned to NAP+SAL or control groups, and treated by investigators blinded to group and genotype from gestational day 8 to 12. This time period was chosen based on previous studies that showed that this is a critical time for VIP action during in utero development [28]. Offspring were weighed and tested from postnatal day (P) 5 to 21 for motor and sensory milestones with standardized tests [7]. The pup’s genotype was determined after completion of all tests. The offspring were tested at 8–10 months of age for learning assessment. The mice were typed for the ability to see as ¼ of these would have carried the rd mutation in a homozygous state and would have been blind. Operators blinded to the offspring’s treatment and genotype performed all the tests.

### Animals

Ts65Dn female mice (The Jackson Laboratory, Bar Harbor, ME) were kept in a 12-hour light/12-hour dark regimen, with food (6% fat diet) and water available at all times. The mice received humane animal care in compliance with the National Institutes of Health (NIH) guidelines for care and use of experimental animals. The protocol was approved by the National Institute of Child Health and Human Development (NICHD) Animal Care and Use Committee. Females were mated with B6EiC3SnFi male mice; the animals were checked twice daily and the day of appearance of a vaginal plug was considered day 0 (E0) of pregnancy.

### Treatment

Ts65Dn (DS) and control pregnant females were treated (intraperitoneal injection) on pregnancy days 8 through 12 (a typical mouse gestation is 18–21 days) with saline (placebo) or peptides (NAP 20 µg +SAL 20 µg) daily. NAP and SAL were obtained from SynPep, Dublin, CA. NAP was diluted in 50 µL of dimethyl sulfoxide and diluted in filtered Dulbecco’s phosphate-buffered saline (DPBS); SAL was dissolved and diluted in filtered DPBS.

### Learning Assessment

The Morris watermaze is a well-established test that evaluates spatial learning, a measure of cognitive function [29]. The apparatus consists of a circular pool with a water level of approximately 30 cm and maintained at 24–26°C. Nontoxic Tempura paint is added to make the water opaque and blend with the colour of the pool wall. External cues (arrow, star, circle, and rectangle) are placed around the pool as a reference for the mice. A transparent platform is placed in the pool and kept in a fixed location throughout the testing period. The platform surface is hidden, submerged under the water surface. Each day, the mouse was positioned on the platform for 15 seconds before being placed into the pool. The mouse was placed into the water at the same location for each trial and allowed to swim freely to find the hidden platform using the external visual cues. On the first day of testing, animals were allowed to remain on the platform for 60 seconds. Each animal underwent 4 consecutive trials daily, with the average time or latency required to find the platform recorded over 5 consecutive days. The animal was given 90 seconds to find the platform, if the animal could not they were rescued and 90 seconds was recorded. Each trial was tracked using an overhead camera interfaced with a computer which recorded the time and path travelled. The latency to find the hidden platform for each trial was recorded and the average of the trials was calculated for each of the five days.

### Genotyping of the Ts65Dn Mice

For genotyping, tail tips (2 mm/sample) from adult mice were collected after the completion of the watermaze. A previously published method was used [23], [30]. All examiners were blinded to the treatment groups and genotype.

### Statistical Analysis

Two-way ANOVA followed by Neuman-Keuls test for multiple comparisons was used for analysis. A P value less than 0.05 was used to denote statistical significance.

## Results

Given the difficulty breeding these animals, the breeding occurred between 2008 and 2010 with four separate watermaze experiments performed as the animals reached the appropriate age for the testing (8–10 months of age). In all, 33 control animals from 9 litters receiving placebo, 30 control animals from 8 litters receiving peptides, 11 DS animals from 4 litters receiving placebo and 10 DS animals from 6 litters receiving prenatal peptides were tested.

**Figure 1:**
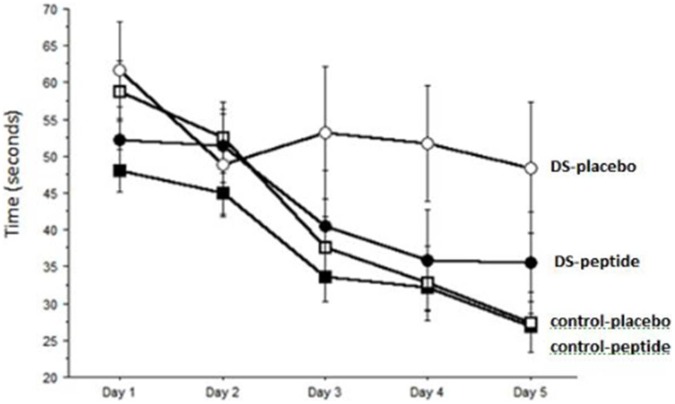
Assessment of spatial learning in the Morris watermaze. Learning was assessed by the latency in seconds to find the hidden platform. Animals with DS who prenatally received saline (placebo, open circles) did not learn over the 5 day period. Control animals (open squares) who prenatally received saline (placebo) did learn with an approximate halving of their latency. Control animals who prenatally received NAP+SAL (closed squares) also learned over the five day period. Animals with DS who prenatally received NAP+SAL (closed circles) learned over the five day period, similar to controls and significantly better than DS-placebo (p<0.01).

Trisomic mice who prenatally received placebo (DS-placebo; n = 11) did not demonstrate learning over the five day period. DS mice that were prenatally exposed to peptides (DS-peptides; n = 10) learned significantly better than DS-placebo (p<0.01), and similar to control-placebo (n = 33) and control-peptide (n = 30) (Figure 1).

## Discussion

Prenatal treatment with the neuroprotective peptides NAP and SAL prevented the learning deficits in adult mice, with learning similar to control animals. Our findings are novel in that we propose the possibility of an intervention during pregnancy that may prevent or reverse the learning deficits of DS. These results extend the benefit shown with prenatal treatment in the Ts65Dn model where NAP and SAL prevented developmental delay in the DS offspring and with postnatal treatment where NAP and SAL prevented learning deficit in Ts65Dn animals treated during adulthood [23], [26]. The results are consistent with studies of learning enhancement with NAP and SAL from our group in a model of fetal alcohol syndrome [25] and in normal animals [31]. Others have also shown that neonatal mice treated with intraperitoneal NAP had increased performance in the Morris watermaze and that VIP is dysregulated in the Ts65Dn partial trisomy model for DS [32], [33].

These results may, at least in part, be explained by our findings of alterations in the N-methyl-D-aspartate (NMDA) receptors. Using this DS mouse model we have shown that prenatal administration of NAP and SAL during pregnancy has long-lasting effects, specifically increasing NR2B and GABAAα3 expression in adult Ts65Dn mice to levels similar to wild-type controls. In the NMDA receptors, the NR2B receptor is the more plastic subunit, thus a decrease in NR2B would make the synapse less plastic. Given the interaction between the GABA and NMDA receptors, a decrease in GABAAα3 would provide less inhibitory tone on the synapse causing overexcitation and ultimately termination of this neural synapse. Restoring the levels of these receptors to the level in controls may in part explain how the peptides NAP and SAL prevent adult learning impairment in the mouse model [34]. Fernandez et al. [35] demonstrated that chronic systemic treatment of Ts65Dn mice with GABAA antagonists causes a persistent post-drug recovery of cognition and LTP. The results support the hypothesis that excessive GABA-mediated inhibition in the Ts65Dn brain actively interferes with declarative memory in Ts65Dn mice [35].

Regarding the protective effects of the peptides on learning and memory, previous studies of hippocampal cultures demonstrated that the peptides alter glutamate release and NMDA receptors [36], both of which are important in learning and memory. Particularly, treatment of hippocampal neurons with SAL controls NR2A and NR2B subunit stability of the NMDA receptor in neurons that have yet to establish efficient synaptic connections [36]. Although the exact mechanism by which SAL influences the synaptogenesis and neurotransmission used by glutamate remains unknown, in vitro studies strongly suggest that the peptide interacts with and regulates the glutaminergic synapses in developing neural systems. Kleschevnikov et al. found that LTP could not be elicited in the dentate gyrus of Ts65Dn mice. They suggested that excessive inhibition of dentate granule cells was shown to restrict synaptic activation of NMDA receptors and to inhibit LTP [37].

One of the neuropathologic characteristics of DS is a glial deficit that likely induces alterations in VIP and its related neuropeptides. In the Ts65Dn mouse, we and others have showen a decrease in ADNP and an upregulation of VIP [20], [23]. Prenatal treatment with NAL and SAL resulted in a normalization of ADNP and of the glial marker GFAP in Ts65Dn adult brains [23]. Treatment with the peptides may have overcome the glial deficit by restoring the appropriate neuropeptides. Nonetheless, treatment did not prevent VIP upregulation; it is possible that other mechanisms regulate VIP release [23].

Previous studies have shown that ADNF and ADNP are neuroprotective in the presence of toxins associated with neurodegenerative disorders, and that neonatal treatment with ADNP attenuated head injury–related dysfunctions in adulthood [38]. Numerous studies have shown the protective effects of VIP-related peptides against alcohol developmental effects [24], [25], [39]–[42]. Here we confirm the role of NAP and SAL in the prevention of learning impairment in a model of DS.

The strengths of this work include the study design and the validation of the results in three different sets of animals. Moreover, this work confirms that the Ts65Dn is a model that is particularly useful in understanding the biological basis for some of the developmental abnormalities and learning deficits of DS subjects.

The high mortality of the DS animals reduced the number of animals available, and consequently the pathway to test to understand the mechanism of action of the VIP related peptides. We encourage further studies to delineate pathways underlying the mechanism behind learning enhancement in the Ts65Dn mouse model of Down syndrome.

### Conclusion

In conclusion, these findings highlight a possibility for the prevention of sequelae in DS and other conditions with learning deficit. Because DS can be diagnosed prenatally, an intervention during pregnancy that may improve cognitive function is an attractive option.
